# Fosmanogepix: A Review of the First-in-Class Broad Spectrum Agent for the Treatment of Invasive Fungal Infections

**DOI:** 10.3390/jof6040239

**Published:** 2020-10-22

**Authors:** Karen Joy Shaw, Ashraf S. Ibrahim

**Affiliations:** 1 Hearts Consulting Group, LLC, Poway, CA 92064, USA; 2 The Lundquist Institute for Biomedical Innovation at Harbor-University of California Los Angeles (UCLA) Medical Center, Torrance, CA 90502, USA; 3 David Geffen School of Medicine at University of California, Los Angeles (UCLA), Los Angeles, CA 90095, USA

**Keywords:** fosmanogepix, FMGX, APX001, manogepix, MGX, APX001A, Gwt1, antifungal, *Candida*, Aspergillus, rare mold

## Abstract

Fosmanogepix is a first-in-class antifungal currently in Phase 2 clinical trials for the treatment of invasive fungal infections caused by *Candida, Aspergillus* and rare molds. Fosmanogepix is the N-phosphonooxymethylene prodrug of manogepix, an inhibitor of the fungal enzyme Gwt1. Manogepix demonstrates broad spectrum in vitro activity against yeasts and molds, including difficult to treat pathogens. Because of its novel mechanism of action, manogepix retains potency against many resistant strains including echinocandin-resistant *Candida* and azole-resistant *Aspergillus.* Manogepix is also active against pathogens that demonstrate intrinsic resistance to other drug classes, such as *Scedosporium, Lomentospora prolificans*, and *Fusarium* with variable activity against Mucorales. Fosmanogepix demonstrates significant in vivo efficacy in mouse and rabbit disseminated infection models due to *C. albicans, C. glabrata, C. auris, C. tropicalis, Coccidioides immitis*, and *F. solani* as well as pulmonary infection models of *A. fumigatus, A. flavus, S.* *prolificans, S. apiospermum* and *Rhizopus arrhizus.* Clinical trials demonstrated high oral bioavailability (>90%), enabling switching between fosmanogepix intravenous and oral formulations without compromising blood levels. Favorable drug-drug interaction, tolerability, and wide tissue distribution profiles are observed making fosmanogepix an attractive option for the treatment of invasive fungal infections. This systematic review summarizes the findings of published data on fosmanogepix.

## 1. Introduction

There is a critical need for new antifungal agents for the treatment of life-threatening invasive fungal infections which have become more common among immunocompromised patients. These include solid-organ or hematopoietic stem cell transplant recipients, cancer chemotherapy patients, or patients with underlying disease that leads to immunosuppression, as well as patients who are critically ill. Global estimates of life-threatening invasive fungal infections exceed 2 million infections per year that include several invasive mycosis infections: candidiasis (>400,000), pneumocystosis (>400,000), aspergillosis (>200,000), cryptococcal meningitis (>1,000,000) [1]. Increased use of antibiotics which disrupt normal bacterial colonization, as well as the use of immunosuppressive drugs have contributed to the higher frequency of these infections [2].

Currently available antifungal agents have one or more liabilities including renal and liver toxicity, treatment spectrum, rising rates of resistance, drug-drug interactions, pharmacokinetic variability and imperfect drug exposure in some infected organs. Amphotericin B (AMB) is only administered intravenously (IV) and demonstrates significant nephrotoxicity [3]. The widely used azoles demonstrate a broad yeast spectrum, efficacy against *Aspergillus* and some Mucorales spp., and are available both IV and orally. However, their use is hampered by liver toxicity, drug-drug interactions, and increased resistance. The azole class is also limited in its spectrum of activity against the rare mold pathogens. According to the Centers for Disease Control and Prevention (CDC), approximately 7% of all *Candida* bloodstream isolates are resistant to fluconazole (FLC), most of which are *C. glabrata* [4]. Similarly, >90% of New York *C. auris* strains are resistant to FLC [5,6]. Among the molds, triazole-resistant *A. fumigatus* are a growing concern due to prolonged azole exposure in patients with chronic pulmonary aspergillosis, or due to environmental exposure of isolates to triazoles used in agriculture [7]. The echinocandins have a more favorable safety profile and are widely used as first-line therapy for *Candida* infections; however, the spectrum of activity is variable against molds, they are only available IV, and do not achieve sufficient drug levels in brain tissue, which can be the site of fungal infections [8]. While echinocandin resistance remains relatively uncommon for most *Candida* species, this is not the case for *C. glabrata*, with rates exceeding 10% at selected institutions [9].

Thus, there are limited options for the adequate treatment of many yeast and mold infections, especially rare molds, which often demonstrate high levels of intrinsic resistance to many antifungal agents. High levels of morbidity and mortality are often observed in invasive fungal infections despite antifungal treatment, and mortality ranges from 46–75% for candidiasis, 30–95% for aspergillosis and 30–90% for rare mold infections [1]. A high percentage of fatalities (>50%) is still observed in invasive pulmonary aspergillosis among severely immunosuppressed patients, such as neutropenic leukemic and transplant patients [10]. Mortality was >50% in patients with invasive scedosporiosis [11] and disseminated *Fusarium* infections [12]. Similarly, the overall mortality rate of mucormycosis is >40% and it approaches 100% in patients with disseminated disease, persistent neutropenia, or brain infection [13,14,15]. Clinical trials for the treatment of these invasive fungal infections are difficult due to the relatively small patient populations, compounded by the limited number of patients who are eligible for clinical trials. Consequently, it is more difficult to guide treatment decisions for these infections.

More favorable clinical outcomes are usually associated with improvement in neutrophil counts, rapid diagnosis of the infecting organism, and the early use of the appropriate antifungal therapy. However, diagnosis of fungal disease is often delayed, and thus the need for a safe and effective broad spectrum agent that can be used empirically are important attributes of any new antifungal agent. Fosmanogepix (FMGX, formerly APX001 and E1211) is a first-in-class N-phosphonooxymethylene prodrug that is rapidly and completely metabolized by systemic phosphatases to the active moiety, manogepix (MGX, formerly APX001A and E1210) [16]. In clinical trials FMGX demonstrated high bioavailability (>90%), and thus both IV and oral formulations are currently being developed.

## 2. Literature Review

PubMed and Embase databases were searched for information on fosmanogepix, APX001, E1211, manogepix, APX001A, and E1210. A total of 38 citations were identified which contained original information on antifungal spectrum, resistance, animal models of infection, and pharmacokinetics/pharmacodynamics. In addition, proceeding from conferences such as International Conference on Antimicrobial Agents and Chemotherapy (ICAAC), European Congress of Clinical Microbiology and Infectious Diseases (ECCMID), and other international symposia identified 13 additional oral and poster presentations of in vitro studies, efficacy studies, chemical properties, DMPK, ADME and clinical trials. Data from abstracts or presentations that were subsequently published or were encore posters were excluded from analysis, as were review articles.

## 3. Discovery of Manogepix

**Figure 1:**
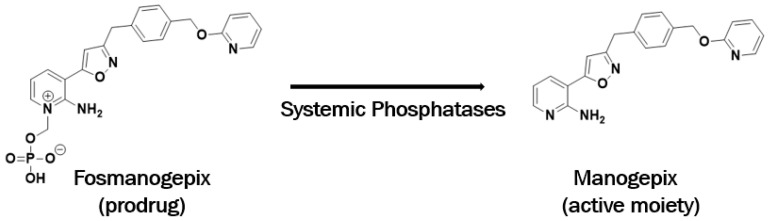
The structures of fosmanogepix and manogepix.

Assembly of mannoproteins into cell wall glucan is a critical and unique feature of fungi. To exploit this pathway as a potential antifungal target, researchers at Eisai Co. utilized an assay to identify small molecules that interfere with cell wall localization of yeast glycosylphosphatidylinositol (GPI)-anchored mannoproteins [17]. As a result of this work, 1-[4 butylbenzyl]isoquinoline (BIQ) was discovered to inhibit both fungal growth and surface expression of GPI-mannoproteins in *Saccharomyces cerevisiae* and *C. albicans*. The investigators identified that the Gwt1 protein was the target of BIQ. Further work by this group and others lead to the search for more potent Gwt1 inhibitors that could be developed as antifungal drugs [18]; and significant lead optimization and understanding of the structure-activity relationship (SAR) [19] culminated in the identification of the preclinical candidate E1210 [20]. This compound was in-licensed by Amplyx Pharmaceuticals in 2015 and was renamed APX001A and then manogepix (Figure 1). The prodrug fosmanogepix (Figure 1) is currently in clinical development for the treatment of *Candida, Aspergillus*, and rare mold invasive infections.

## 4. Mechanism of Action

**Figure 2:**
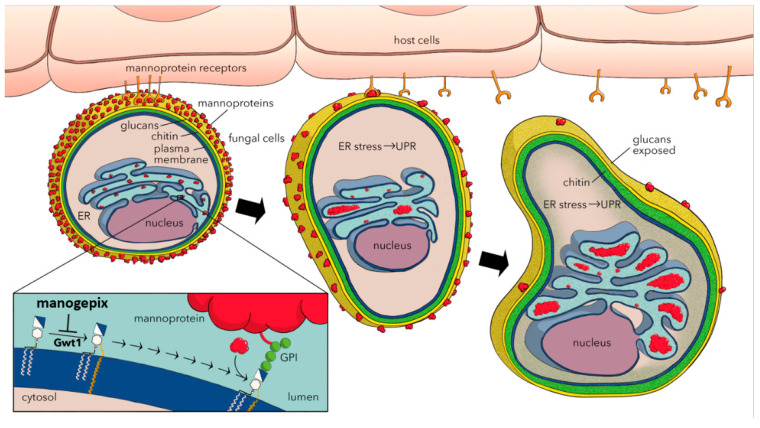
The mechanism of action of manogepix. Modified from 2019 Covel et al. [26]. Gwt1 is an enzyme which catalyzes an acyl group transfer reaction, an essential part of the GPI biosynthesis pathway. This occurs in the lumen of the endoplasmic reticulum. Gwt1 catalyzes the inositol acylation of glucosaminyl phosphatidylinositol (GlcN-PI), resulting in the formation glucosaminyl(acyl)phosphatidylinositol (GlcN(acyl)PI), an intermediate in the biosynthesis of GPI anchors.

MGX is a first-in-class antifungal that inhibits the fungal Gwt1 protein, a conserved enzyme that catalyzes inositol acylation, an early step in the GPI-anchor biosynthesis pathway [21,22] (Figure 2). Gwt1 is essential for trafficking and anchoring mannoproteins to the cell membrane and outer cell wall [17], and inhibition of this enzyme in both *C. albicans* and *S. cerevisiae* has been demonstrated to affect maturation and localization of GPI-anchored mannoproteins. Since these mannoproteins are required for cell wall integrity, adhesion, pathogenicity, and evading the host immune system, inhibition of Gwt1 by MGX has many physiological effects [21,23,24]. Similar to other Gwt1 inhibitors such as gepinacin, these effects include reduction in cell wall-linked mannoproteins, reduction in hyphal formation, reduction in biofilm formation, malformation of cell size and shape, exposure of the glucan layer and endoplasmic reticulum stress [25] (Figure 2). Importantly, the closest mammalian ortholog, PIGW, is not sensitive to inhibition by MGX [21].

## 5. Effects on Virulence Factors and Biofilms

The effect of MGX on *C. albicans* virulence factors was examined in a study by investigators at Eisai Co. [21]. The cell surface expression of the GPI-anchored Als1 adhesin protein was shown to be inhibited by MGX in a concentration-dependent manner by 44%, 58%, and 74% at concentrations of 0.008 (1 × MIC), 0.03 (4 × MIC), and 0.13 µg/mL (16 × MIC), respectively [21]. This is in contrast to FLC, which showed no effect, and micafungin (MCF), which enhanced Als1p cell surface expression. These data are consistent with the mechanism of action of MGX, which is inhibition of the maturation of GPI-anchored cell surface proteins.

Other virulence factors including adherence and germ tube formation were also examined [21]. MGX inhibited the adherence of *C. albicans* cells to polystyrene with an IC_50_ of 0.0039 µg/mL, two-fold below the MIC of 0.008 µg/mL, while the AMB IC_50_ was ~ 5-fold higher than its MIC. FLC showed no inhibition and MCF enhanced *C. albicans* adherence about 2-fold at concentrations above its MIC value [21]. *C. albicans* germ tube formation was inhibited by MGX, MCF, AMB with IC_50_ values of 0.0071, 0.015, and 0.24 µg/mL, respectively. MGX, MCF and AMB all inhibited germ tube formation at concentrations near their respective MIC values, whereas FLC did not demonstrate inhibition in this assay at concentrations ≤8 µg/mL [21]. The effect of MGX on hyphal growth was evaluated by embedding cells within a matrix and following growth. MGX suppressed hyphal growth at 0.002 µg/mL, and inhibited both hyphal and colony growth at 0.008 µg/mL (1 × MIC).

Finally, the inhibition of biofilm formation by MGX was evaluated in an assay that assessed biofilm density after 24 h of growth, using safranin staining of extracellular polymeric materials [21]. MGX demonstrated an IC_50_ of 0.0044 µg/mL and entirely inhibited biofilm formation at a concentration of 0.008 µg/mL, whereas MCF and AMB IC_50_ values were 0.014 µg/mL and 0.085 µg/mL, respectively. Although FLC showed some inhibition of biofilm formation, this inhibition was not complete at 16 × MIC. The activity of MGX was also examined against sessile *C. albicans* cells. The SMIC_90_ of MGX was 8-fold higher for sessile cells than planktonic cells (0.13 µg/mL and 0.016 µg/mL, respectively), similar to MCF and AMB which also demonstrated an 8-fold increase in the MIC_90_. FLC was not active against sessile cells (SMIC_90_ >16 µg/mL). The activity of MGX was not evaluated against preformed biofilms.

The sum of these data suggests that inhibition of Gwt1 by MGX results in pleiotropic effects on the fungal cell by preventing the maturation of GPI-anchored proteins. MGX has been shown to inhibit a number of important *C. albicans* virulence factors that include cell surface proteins which affect adherence, hyphal formation and biofilm formation. The inhibition of fungal virulence as well as subsequent cell wall damage are likely contributing to the overall activity of this new antifungal agent.

## 6. Time Kill and Post Antifungal Effects

The inhibitory effects of MGX on the growth of *C. albicans* was examined in a time kill experiment. Both MGX and FLC were fungistatic over the 24 h period, and inhibited growth in a concentration-dependent manner [20]. The post antifungal effect (PAFE) of MGX was evaluated both in vitro and in vivo [27]. *C. albicans* was exposed to MGX for 3 h at concentrations of 4 × MIC, 16 × MIC, and 64 × MIC and in vitro PAFE values were determined to be 1.8, 3.9, and 4.6 h, respectively. Using a *C. albicans* neutropenic mouse model, a single oral dose of vehicle, 10 mg/kg or 40 mg/kg MGX was administered 2 h after infection, and colony forming units (CFU)/g kidney were evaluated in mice that were sacrificed during the course of the 36 h experiment. The in vivo PAFE was calculated as the time necessary for *C. albicans* to grow 1 log_10_ CFU/g kidney. MGX administered at 10 mg/kg or 40 mg/kg prolonged the suppression of growth for 14 and 11 h, respectively [27]. The in vivo PAFEs in mice were observed about 10 h after serum drug levels fell below the MIC. This long in vivo PAFE may contribute to the clinical efficacy of FMGX by suppression of organism regrowth during drug trough levels.

## 7. MGX In Vitro Activity against Yeasts

The in vitro activity of MGX against diverse fungal species has been summarized in several publications [26,28,29]. Table 1 summarizes the results of additional studies, including data where >10 isolates per yeast species were examined. The studies include data from the 2017 SENTRY worldwide surveillance study [30], country specific laboratory surveillance data (Spain, Denmark) [29,31] and other collections [32,33,34,35]. Several studies have been conducted comparing MIC or minimum effective concentration (MEC [defined as the lowest drug concentration resulting in the production of small, rounded, compact hyphal forms]) values using both CLSI and EUCAST methodologies and a high degree of essential agreement has been observed. Where differences are observed, CLSI MIC or MEC values are generally 1–2-fold lower than EUCAST values [31,36,37]. Similar to the echinocandins, endpoints are read at 50% inhibition at 24 h for *Candida* and most other yeasts [38], with MEC values determined at 48 h for most of the molds [39].

MGX was highly active against most *Candida* species tested, with MIC_90_ values for key species ranging as follows: *C. albicans* (0.008–0.06 µg/mL), *C. auris* (0.03 µg/mL), *C. dubliniensis* (0.008 µg/mL), *C. glabrata* (0.06–0.12 µg/mL), *C. tropicalis* (0.03–0.06 µg/mL), and *C. parapsilosis* (0.015–0.06 µg/mL) (Table 1). Among the yeasts, exceptions include *C. krusei* and to a lesser extent *C. kefyr* [34] where the MIC_90_ values were higher (≥ 0.5 μg/mL) (Table 1). At least for *C. krusei*, the MIC difference is likely not Gwt1 target-based, but rather due to differences in *C. krusei* cell permeability or efflux. This is based on the finding that isogenic *S. cerevisiae* strains expressing heterologous *C. albicans, S. cerevisiae* or *C. krusei* proteins demonstrated similar MIC values (within 2–fold), unlike *S. cerevisiae* strains expressing the human PIGW protein which, due to target-based differences, resulted in an ~250–fold increase in MIC [42].

Since *C. auris* was not identified in SENTRY surveillance studies until 2017 and less than 10 isolates were observed, a separate review of the activity of MGX, as measured by CLSI methodology, against *C. auris* is shown in Table 2. The CDC collection was comprised of 100 geographically distinct isolates of *C. auris* that included the 4 known international clades [41]. This collection included 6 isolates with elevated MICs to one or more echinocandins, 24 isolates with elevated MICs to AMB, and 2 isolates that were resistant to all three antifungal drug classes. For this collection, the MIC of MGX ranged from < 0.0005–0.015 µg/mL whereas the MICs of the echinocandins ranged between 0.03 to >8 µg/mL for MCF, 0.03 to > 16 µg/mL for caspofungin acetate (CAS), and 0.125 to > 16 µg/mL for anidulafungin (AFG) [41]. Other studies examined both worldwide and country or state specific isolates [35]. Arendrup et al. [37] evaluated 122 strains using both CLSI and EUCAST methodologies and found good essential agreement between the data, although they observed a systematic difference whereby CLSI MICs were generally 1 to 2-fold dilution lower than EUCAST MICs. In addition, the MGX wild-type upper limit (WT-UL)−97.5% and WT-UL-99% were determined using CLSI/EUCAST methodologies: 0.03/0.125 µg/mL and 0.06/0.125 µg/mL, respectively [37]. A recent study by Zhu et al. [35] evaluated the activity of MGX against 200 recent *C. auris* isolates from a New York outbreak where the MIC_90_ was 0.03 µg/mL. All isolates were within the population of wild-type (WT) strains where 0.06 µg/mL defined the WT-UL. MGX was 8- to 32-fold more active than the echinocandins, 16- to 64-fold more active than the azoles, and 64-fold more active than AMB [35]. Importantly, MGX maintained activity vs. echinocandin-resistant, AMB-resistant, and isolates target-based (*ERG11* [lanosterol 14-α-demethylase]) azole-resistant *C. auris* strains. In all studies, MGX demonstrated the lowest *C. auris* MIC_50_ and MIC_90_ values vs. competitor agents (Table 2).

## 8. MGX In Vitro Activity against Molds

MGX demonstrates potent activity against *Aspergillus* and some species of rare molds. The SENTRY Antimicrobial Surveillance Program evaluated the activity of MGX against 1706 contemporary, world-wide clinical fungal isolates collected in 2017 [30]. MGX demonstrated potent in vitro activity against recent fungal isolates, including echinocandin- and FLC-resistant strains [30]. Other laboratories examined more regional organisms, including itraconazole (ITC) resistant strains and cryptic *Aspergillus* species. Using EUCAST methodology, a study of contemporary (2016–2017) clinical isolates from Denmark, showed that MEC_50_/MEC_90_ values for all *Aspergillus* spp. ranged between 0.03–06/0.06–0.125 µg/mL and MEC values were unaffected by ITC-resistance [29].

The data for *Aspergillus* spp. is summarized in Table 3, and MEC_90_ values for MGX ranged from 0.015 to 0.06 μg/mL: *A. fumigatus* (0.03–0.06 μg/mL), *A. flavus* (0.015–0.03 µg/mL), *A. niger* (0.015–0.03 µg/mL), *A terreus* (0.03–0.06 µg/mL). A study of clinical isolates from patients in Spain (2000–2016) showed that MGX was also active against cryptic *Aspergillus* species, with CLSI MEC_90_ values ranging between 0.03–0.06 μg/mL for *A. lentulus, A. fumigatiaffinis, A. udagawae, A. calidoustus* and *A. alliaceus*, whereas posaconazole (POS) MEC_90_ values were 1, 1, 0.5, 16 and 2 µg/mL, respectively [31]. Of note, MGX retained activity against *A. alliaceus* and *A. calidoustus*, which demonstrated MIC_90_ values of 32 µg/mL for AMB, and 16 µg/mL for POS, respectively (Table 3). The MGX MEC_90_ value for *A. thermomutatus* was 4-fold higher than other *Aspergillus* species (0.25 µg/mL).

MGX also demonstrated significant activity against *Fusarium* spp., *Scedosporium* spp. and some fungi from the Mucorales order (Table 3). The MEC_90_ values for MGX against *S. apiospermum, S. prolificans (Lomentospora prolificans), F. solani* and *F. oxysporum* and *G. fujikuroi* were 0.12, 0.12, 0.06, 0.25 and 0.12 μg/mL, respectively in the study conducted by Castanheira et al. [36], which examined a worldwide collection of isolates. These rare mold species were most often highly refractory to all other comparator agents tested. A study of a collection of isolates from Spain indicated that *Fusarium* and *Scedosporium* demonstrated a wider range in MEC values, with a corresponding rise in MEC_90_ values [31] (Table 3). Against *L. prolificans* the CLSI MEC_90_ of MGX was 0.06 µg/mL, using CLSI methodology, in contrast to MICs of 8 and 16 µg/mL for AMB and POS, respectively [31] (Table 3). Although the study of Spanish isolates showed that *Fusarium* CLSI MEC_90_ values for MGX were > 8 µg/mL, low MEC values (< 0.12 µg/mL) were observed for 4 of 10 *F. verticillioides* and 8 out of 10 *F. oxysporum* isolates (MEC_50_ = 0.015 µg/mL) suggesting that some of the strains may be clinically treatable. POS MIC_50_/MIC_90_ values for these strains were >8/ > 8 and 8/ > 8 µg/mL, respectively.

The composite data in Table 3 shows that *Fusarium* spp. MIC_90_ value ranges for azoles were variable or high: ITC, >8 µg/mL; VRC, 4 to >8 µg/mL; and POS, 2 to 16 µg/mL. Similarly, azoles MIC_90_ values were variable or high for *Scedosporium*: ITC, 4 to >8 µg/mL; VRC, 1 to >8 µg/mL; and POS, 2 to 16 µg/mL [31,36]. In a recent study of 49 *F. oxysporum* and 19 *F. solani* species complexes isolates, MGX MEC values were between <0.015–0.03 µg/mL and <0.015 µg/mL, respectively. However, the AMB MIC values ranged between 1–4 and 0.25–4 µg/mL for *F. oxysporum* and *F. solani*, respectively [45]. Thus, the extended spectrum of MGX is notable for its potency against many of the less common but antifungal-resistant fungi *Fusarium* spp. and *Scedosporium* spp.

Activity against 7 species from the Mucorales order was also investigated [31]. In general, high MEC_50_/MEC_90_ values (in µg/mL) were observed: *R. arrhizus* >8/ > 8, *R. microsporus* 4/ > 8, *R. pusillus* > 8/ > 8, *Lichtheimia ramosa* 8/ > 8, *L. corymbifera* >8/ > 8, *Mucor circinelloides* 4/8, and *Cunninghamella bertholletiae* > 8/ > 8. However, 3 of 10 strains of *M. circinelloides* demonstrated CLSI MEC values ≤ 2 µg/mL. These data are similar to the findings of 41 Mucorales (*Mucor, Rhizomucor, Rhizopus, Lichtheimia*, and *Synsephalastrum*) isolated between 2017 and 2019 with MEC values ranging between 0.5 to > 8 µg/mL [28,30]. Although MGX MECs for Mucorales are variable and generally higher than for the other rare molds, in vivo efficacy has been described in two mouse models of mucormycosis using strains with MEC values of 0.25 µg/mL and 4.0 µg/mL [46]. These data suggest that some of the Mucorales with lower MEC values may be clinically treatable.

The composite summaries in Table 1, Table 2 and Table 3 show that MGX demonstrates potent broad-spectrum activity against *Candida, Aspergillus* and other difficult to treat rare molds. In addition, MGX was highly active against *Coccidioides* spp., with an MEC_90_ of 0.008 µg/mL [47].

## 9. Resistance

The potential for development of resistance to MGX was investigated in *C. albicans*, *C. glabrata*, and *C. parapsilosis* by evaluating spontaneous mutation frequencies [42]. A large plate format method was utilized to allow assessment of an inoculum of 1 × 10^8^ CFU/mL. Median spontaneous mutation frequencies for MGX ranged from 3.25 × 10^−8^ (*C. albicans)* to <1.88 × 10^−8^ (*C. glabrata* and *C. parapsilosis*). These data are similar to the spontaneous resistance frequency of AFG, CAS and the experimental drug rezafungin (CD101), which were evaluated using a similar methodology [48].

Serial passage experiments performed using a gradient plate method showed that MIC values increased 8-fold from 0.016 to 0.125 µg/mL for both *C. parapsilosis* and *C. albicans*, however this occurred after passages 3 and 18, respectively [42]. No increase in MIC (≤ 2-fold) was observed for *C. glabrata*. To further explore the potential for resistance development, the broth macrodilution serial passage method was used for *C. glabrata, C. tropicalis* and *C. auris*. However, MIC values of MGX did not increase substantially (≤ 2-fold) for these three *Candida* isolates [42].

To understand the underlying resistance mechanisms, the *gwt1* target gene was sequenced in strains demonstrating increased MIC values and compared to the sequence of the respective wild-type starting strains. A valine to alanine mutation at position 163 (V163A) in the Gwt1 protein was identified in four *C. glabrata* mutants and the corresponding valine to alanine mutation at position 162 (V162A) was also identified in a heterozygous *C. albicans* mutant. A *C. glabrata* V163A Gwt1 mutant was generated using CRISPR and showed similarly reduced susceptibility. These data suggest the importance of this valine residue to MGX binding to Gwt1 orthologs across different species. MGX MIC values of all of these mutants increased 16–32-fold vs. the isogenic parent strain. However, no changes in MIC values were observed for AMB, CAS, or FLC, demonstrating a lack of cross-resistance.

Two *C. parapsilosis* and *C. albicans* mutants were identified that demonstrated 4- to 8-fold decreased susceptibility to MGX in which the *gwt1* gene sequence was similar to the wild-type [42]. These mutants were further investigated and the change in MIC was determined to be efflux-mediated [49]. The *C. parapsilosis* mutant contained a mitochondrial deletion, which activated expression of the major facilitator superfamily transporter gene *MDR1*. The *C. albicans* mutant demonstrated a gain-of-function mutation in the transcription factor gene *ZCF29*, which activated expression of the ATP-binding cassette transporter genes *CDR11* and *SNQ2*. These two mutants also showed 2- and 4-fold decreased susceptibility to FLC, but not AMB or CAS [42,49]. These data are consistent with the observation that a subset of FLC resistant mutations in several species may lead to small increases in MGX MIC values [50]. Given the low MGX MIC values of the starting strains and that the two mutant strains demonstrated MIC values ≤ 0.056 µg/mL, these individual mutations may not result in clinically significant resistance and the strains may remain in the clinically treatable range.

## 10. Activity against Echinocandin-, Azole- and AMB-Resistant *Candida* spp.

Several studies have examined the activity of MGX against collections of azole-, echinocandin- or AMB-resistant mutants. Pfaller et al. examined 20 FLC-resistant *Candida* spp. and 15 CAS-resistant *Candida* spp., and no increase in MGX MIC was observed [32]. Similarly, Miyazaki et al. [20] reported no cross-resistance to azoles when the MGX MIC_90_ values of a collection of 140 FLC-susceptible *Candida* spp. was compared to 18 FLC-resistant *Candida* spp. (MIC_90_ 0.06 vs. 0.03 µg/mL, respectively).

The activity of MGX has also been evaluated against specific target-based resistant fungi including those that are echinocandin-resistant (*fks1, fks2*) or azole resistant (*erg11*). Zhao et al. compared the MGX MIC of 3 *C. glabrata* strains which were wild-type for Pdr1 and Fks1/2, echinocandin resistant (Fks1-S629P) or multidrug resistant (Pdr1-G1079R, Fks2-S663P) [51]. The MGX MIC values for the three strains were 0.03, 0.03 and 0.125 µg/mL, respectively, suggesting that the MGX MIC value was elevated 4-fold in the MDR strain in which there is a higher expression of efflux pumps due to the Pdr1-G1079R mutation, but not the echinocandin-resistant strain [51,52]

In a study of *C. albicans* isolates with known *fks1* mutations or FLC-resistance, MIC values were unchanged vs. the susceptible control strains (≤ 0.03 µg/mL) [40]. As part of the 2017 SENTRY surveillance study that included 1340 *Candida* isolates, the MIC values of 10 *C. glabrata* and 3 *C. albicans* strains with mutations in *fks1* or *fks2* were also assessed [30]. MGX showed good activity against both echinocandin-susceptible and echinocandin-resistant *Candida* isolates. For the *C. glabrata* echinocandin-resistant strains, the MGX MIC values ranged between 0.016–0.12 µg/mL, all below the WT-UL cutoff value of ≤ 0.25 µg/mL. The MGX MICs of the three echinocandin-resistant *C. albicans* strains were 0.008 µg/mL, again below the WT-UL of ≤ 0.03 µg/mL. Similarly, a recent study examined 17 *Candida* isolates with *fks* target gene-encoded hotspot alterations and found that MGX activity against these isolates was similar to wild-type strains, with the exception of two *C. albicans* with MGX MICs of 0.125 and 0.25 µg/mL which were also pan-azole resistant [34]. The SENTRY 2018 and 2019 study also evaluated 8 *C. glabrata* strains with *fks1* and/or *fks2* mutations, and found that MGX MICs ranged between 0.008–0.12 µg/mL, values that are ≤ 0.12 µg/mL, the WT-UL determined from the collection of 460 *C. glabrata* isolates [28]. Thus, no cross-resistance with echinocandins was observed.

In some of the studies where MGX was evaluated against resistant clinical isolates, the resistance mechanisms were not defined genetically. These studies included *C. auris* isolates that were resistant to azoles, echinocandins and/or AMB [35,37,41], and other FLC or CAS-resistant *Candida* spp. [32]. For the *C. auris* isolates, Arendrup et al. [37] examined the correlation between MGX MICs and other antifungal drugs in a study of 122 isolates from India. No correlation was observed between the MIC values of MGX and AMB, MFG or AFG, consistent with the unique mechanism of action of MGX. However a correlation was observed between MGX and the azoles (CLSI methodology): FLC (*p* < 0.001), VRC (*p* = 0.017), isavuconazole (ISA) (*p* = 0.017) and ITC (*p* = 0.016), although the mechanism behind the azole resistance was not characterized in these isolates and the impact on MGX clinical efficacy is unclear.

Despite the fact that FLC and MGX target different fungal enzymes (Erg11 and Gwt1, respectively), a correlation between increased MGX and FLC MIC values was first identified in a study that examined 540 yeast bloodstream isolates from the nationwide Danish surveillance program (2016 and 2017 isolates) along with a collection of 122 clinical isolates of *C. auris* from tertiary care hospitals in India (2010 to 2015) [50]. Single center WT-UL were determined, and 4 of 540 bloodstream isolates (1 *C. dubliniensis*, 2 *C. glabrata*, 1 *C. tropicalis*) were identified with MIC values that exceeded the WT-UL (0.03, 0.25, 0.125 µg/mL, respectively). All four strains were FLC resistant as well (32, > 32, ≥ 16 µg/mL, respectively). However, many FLC-resistant isolates did not show elevated MGX MICs, suggesting that only a subset of FLC-resistant mechanisms may affect MGX susceptibility. In addition, a linear correlation between modal MICs of FLC and MGX was observed for several species including *C. neoformans, C. albicans*, *C. dubliniensis*, *C. glabrata*, *C. tropicalis*, but not *C. auris* or *C. guilliermondii* where MGX modal MICs were low (0.008–0.016 µg/mL) but FLC modal MICs were high (4 µg/mL and 512 µg/mL, respectively) [50].

A later study by the same investigators examined a collection of 835 yeast isolates received at the reference laboratory in Denmark during 2018 [34]. The MGX WT-UL was determined for species in this collection, and WT populations of 16/20 yeast species were highly susceptible with modal MICs from 0.004–0.06 µg/mL. Further examination of 5 species with ≥ 10 isolates (*C. albicans, C. dubliniensis, C. glabrata, C. parapsilosis* and *C. tropicalis*) demonstrated that 3% (11 of 364 isolates) had MGX MIC values above the WT-UL defined for the species. Of the 11 isolates, 9 also demonstrated MIC values above the FLC tentative EUCAST ECOFF values. In contrast, 40 strains with FLC MIC values above the EUCAST ECOFF were within the WT population of MGX MIC values. These data are consistent with the hypothesis of a linkage between MGX and FLC MIC values in a small subset FLC-resistant strains. These data may be explained by the analysis of laboratory generated mutants in which some isolates with efflux related mutations displayed a 4- to 8-fold increase in the MIC of MGX and a concomitant 2- to 4-fold increase in the FLC MIC [49]. This is in contrast to target-based Gwt1 mutants in which increased MIC values for MGX, but not FLC, were observed [42]. Further studies are necessary to understand the impact of target-based vs. efflux-based mechanism(s) in clinical isolates, as well as their impact on FMGX efficacy.

## 11. Activity against Azole- and AMB-Resistant *Aspergillus* spp.

The activity of MGX vs. resistant molds has also been investigated including AMB-resistant *A. terreus* and ITC-resistant *Aspergillus* [44]. MEC values for these strains were within or below the range of values observed for the larger collection of *Aspergillus* strains (0.015–0.06 µg/mL) suggesting a lack of cross-resistance. Arendrup et al. examined the activity of MGX against a collection of 161 clinical isolates of *Aspergillus* and other molds from Denmark [29]. Ten *A. fumigatus* isolates were ITC-resistant and harbored a variety of Cyp51A mutations. MGX demonstrated equivalent activity against both the resistant (GM-MEC: 0.056 µg/mL, range 0.03–0.125 µg/mL) and susceptible *A. fumigatus* strains in this collection (GM-MEC 0.053 µg/mL, range 0.016–0.125 µg/mL). Similarly, Huband *et al.*, [28] evaluated 10 *A. fumigatus* azole non wild-type isolates from around the world, 8 of which carried mutations in Cyp51A or Cyp51B. MGX MECs for the 10 strains ranged between 0.008–0.015 µg/mL, all below the WT-UL of *A. fumigatus* determined from the collection of 397 worldwide *A. fumigatus* isolates.

Overall, no cross-resistance was observed, with the exception of the increase in MGX MIC values in a small number of FLC-resistant *Candida* strains, which is likely to be efflux-based.

## 12. Pharmacokinetics/Pharmacodynamics

Definitive pharmacokinetic/pharmacodynamic (PK/PD) studies were conducted to evaluate the driver for efficacy using a well-validated disseminated candidiasis model. The results of this analysis demonstrated that free fraction area under the plasma concentration-time curve over MIC (*f*AUC/MIC) and free fraction maximum (peak) plasma drug concentration over MIC (*f*C_max_/MIC) were strongly correlated to efficacy (R^2^ values 0.88 and 0.83, respectively) [50]. PK/PD studies using this model of disseminated *C. albicans* (5 strains), *C. glabrata* (5 strains) and *C. auris* (4 strains) infections showed that the median free drug AUC/MIC PK/PD targets for stasis for these 3 species were 191.59, 11.48, and 99.69, respectively (D. Andes, personal communication) [50]. Definitive PK/PD experiments were also performed in a neutropenic, corticosteroid treated murine model of invasive pulmonary aspergillosis (IPA) against 6 *A. fumigatus* clinical strains (3 wild-type [WT], 3 Cyp51 mutants) [51]. Similar to the *Candida* study, *f*AUC/MEC was demonstrated to strongly correlate with efficacy. Exposure-response relationships for all strains were similar and the median free drug AUC/MEC PK/PD targets for stasis was 47.6 [51].

## 13. In Vivo Efficacy

Earlier studies conducted by Eisai Co. evaluated the efficacy after administration of the active moiety, MGX, rather than the prodrug. Reduction in CFU was observed in the oropharyngeal candidiasis model, and increased survival was observed in models of disseminated candidiasis, pulmonary aspergillosis and disseminated fusariosis as summarized in Table 4 [53]. Eisai Co. also examined the effect of the co-administration of FMGX and echinocandins in an *A. flavus* pulmonary infection model where mice were immunosuppressed with 200 mg/kg of 5-FU given 5 days prior to infection, treatment was administered Day 0–3, and Day 14 survival was evaluated [53]. The combinations of FMGX plus MFG or FMGX plus CAS significantly increased survival over the administration of a single agent. A caveat to this study was that both FMGX and the echinocandins were administered at doses in mice that give rise to exposures well below what is achieved clinically. Thus, it is not clear whether combination dosing in humans at approved dosing regimens would recapitulate these findings and improve patient outcome over monotherapy.

The prodrug FMGX has been examined in a wide variety of animal models of invasive fungal infections including disseminated models due to *C. albicans, C. glabrata, C. auris, C. immitis, C. neoformans, F. solani* infections, and pulmonary models of *A. fumigatus, A. flavus, S. prolificans, S. apiospermum* and *R. arrhizus* which are summarized in Table 4. All of the models utilized mice, with the exception of a rabbit model of *Candida* endophthalmitis and hematogenous meningoencephalitis [55]. In addition to demonstration of increased survival, several of the models demonstrated reduction in fungal burden in lung, kidney, spleen, eye, and brain after FMGX administration. This latter is particularly important in some invasive infections given that the echinocandins do not show significant efficacy in brain infections. The results of these models are summarized below.

In addition to the PK/PD study conducted with *C. albicans, C. glabrata* and *C. auris* [54], the efficacy of FMGX was evaluated in two other disseminated *C. auris* models which examined both survival and reduction in fungal burden. In the first model, 90–100% survival and significant reductions in CFU in kidney, lung, and brain were observed when 78 mg/kg BID or 78 mg/kg thrice daily (TID) was administered intraperitoneally (IP) [33]. In a second *C. auris* model, treatment was delayed for 24 h post-inoculation [43]. Significant improvements in survival at Day 21 were observed in each group administered FMGX (104 and 130 mg/kg IP twice daily [BID] or 260 mg/kg IP BID) and CAS (10 mg/kg IP once daily [QD]); however FLC (20 mg/kg administered orally [PO], QD) was not effective in this model [43]. Significant reductions in kidney fungal burden vs. control were observed with 260 mg/kg BID FMGX (3.86 log_10_ CFU/g) or with 10 mg/kg CAS (3.41 log_10_ CFU/g). Significant reductions were also observed in brain in mice treated with 260 mg/kg BID FMGX (2.99 log_10_ CFU/g) compared with that of the vehicle control. In contrast, brain fungal burden was not significantly reduced in mice treated with the lower doses of FMGX, FLC, or CAS [43].

The efficacy of FMGX was examined in a disseminated rabbit model of *Candida* endophthalmitis and meningoencephalitis where antifungal therapy was initiated 48 h after inoculation and continued throughout the course of the experiments for 7 days [55]. Rabbits treated with FMGX at 25 mg/kg BID, 50 mg/kg BID, and 100 mg/kg BID demonstrated quantitative clearance of *C. albicans* from tissues including cerebrum, cerebellum, spinal cord, cerebrospinal fluid (CSF), meninges, and aqueous humor.

The in vitro and in vivo activity of MGX/FMGX was evaluated against *Cryptococcus* [55]. The MIC_90_ of 9 strains of *C. neoforma*ns and 9 strains of *C. immitis* was 0.5 µg/mL, indicating that MGX was less potent against *Cryptococcus* spp. than *Candida* spp. The efficacy of administration of FMGX, FLC, or the combination of FMGX and FLC was evaluated in a disseminated mouse model of cryptococcal meningitis using strain H99 (H99 MGX MIC = 0.25 µg/mL). In this model, MGX exposures were similar to those obtained in Phase 1 clinical studies, and the reduction in fungal burden in brain tissue for FMGX and FLC was 0.78 and 1.04 log_10_ CFU/g, respectively whereas the combination resulted in a reduction of 3.52 log_10_ CFU/g, suggesting possible additivity brain tissue [56]. However, in lung tissue, no statistically significant differences between the treatment groups was observed.

The PK of MGX after oral administration of FMGX has been evaluated in mice, and the half-life was determined to be 1.4–2.75 h, which is significantly shorter than what has been observed in healthy human volunteers during Phase 1 clinical studies (2 to 2.5 days) [54,63,64]. As a result, BID, TID, or more frequent dosing intervals have been utilized in order to achieve efficacy in mouse models. To counter this rapid metabolism in mice, oral administration of 1-aminobenzotriazole (ABT), a non-specific inhibitor of cytochrome P450 enzymes, 2 h prior to each FMGX dose was shown to increase the MGX half-life in mice to approximately 6–9 h and greatly enhanced exposures. Several studies conducted with different fungi showed that there is no antifungal effect of ABT alone either in vitro or *in vivo*, and that there was no synergy between ABT and antifungal agents in vitro or in vivo [51,59]. Thus, pretreatment with 50 mg/kg ABT 2 h prior to FMGX was utilized as a standard dosing regimen for several efficacy experiments where 78 mg/kg FMGX plus ABT gives rise to MGX exposures similar to what was observed in healthy human volunteers.

Subsequent efficacy experiments in mice have shown that once daily dosing of FMGX in conjunction with ABT results in improved efficacy as demonstrated by reduction in fungal burden, increased survival, and histological improvement. In invasive candidiasis, FMGX was shown to be an effective antifungal agent for the treatment of susceptible, echinocandin-resistant, or MDR *Candida* infections. Administration of 26 mg/kg plus ABT sterilized kidneys in mice infected with *C. albicans*, while FMGX alone at the same dose resulted in a modest fungal burden reduction of only 0.2 log_10_ CFU/g, relative to the vehicle control [51]. In the presence of ABT, 2 days of once-daily dosing with FMGX at 26 mg/kg also demonstrated significant in vivo efficacy in the treatment of *C. glabrata* infections in mice [51]. Potent kidney burden reduction was achieved in mice infected with susceptible, echinocandin-resistant, or multidrug resistant strains. In contrast, the standard of care MFC was ineffective in treating infections caused by the resistant *C. glabrata* isolates.

In a pulmonary coccidioidomycosis model where treatment was initiated one week after infection, treatment of mice with 26 mg/kg FMGX (QD, with ABT) or FLC (25 mg/kg BID) reduced log_10_ CFU in the lung by >2.5 fold (*p* < 0.001) versus the ABT control and completely prevented dissemination to the spleen [47]. The FMGX treated mice also demonstrated significantly longer survival than control or FLC-treated mice.

The efficacy of FMGX in conjunction with ABT was examined in an invasive pulmonary aspergillosis (IPA) model in mice infected with *A. fumigatus* [59]. This model utilized a severely immunocompromised mice in which the cyclophosphamide/cortisone acetate treatment results in pancytopenia for at least 9 days from the first administered dose. Treatment of mice with FMGX at 78 mg/kg QD, 78 mg/kg BID, or 104 mg/kg QD significantly enhanced median survival time and prolonged Day 21 post-infection overall survival when compared to placebo. Furthermore, administration of FMGX resulted in a significant reduction in lung fungal burden [4.2 to 7.6 log_10_ conidial equivalents (CE)/gram tissue] vs. the untreated control and resolved the infection as judged by histopathological examination. The observed survival and tissue clearance were comparable to a POS dose that was at least twice as high as the POS dose required in mice to achieve an AUC consistent with efficacy in the clinic [65].

Galactomannan (GM) detection in biological samples has been shown to predict therapeutic response by azoles and polyenes [66]. GM was evaluated as a biomarker of FMGX efficacy in the IPA model, as described [60]. Cohorts of mice were sacrificed at 48 h, 72 h, and 96 h to evaluate changes in CFU/g of lung tissue as well as serum GM and bronchoalveolar lavage (BAL) GM levels from the same mice. FMGX or POS treatment resulted in a ~6–7 log reduction in CE/g lung tissue after 96 h versus placebo. Changes in GM levels in BAL and serum mirrored reductions in lung CE with significant decreases seen after 96 h or 72 h for FMGX or POS, respectively, suggesting the potential use of GM as a biomarker of FMGX efficacy in immunosuppressed mice.

The efficacy of FMGX was also evaluated in highly immunosuppressed murine models of disseminated fusariosis and pulmonary scedosporiosis [61]. In the pulmonary scedosporiosis model, *S. apiospermum* DI16–478, which is susceptible to azoles as well as to MCF, was utilized and treatment was initiated 16 h post infection. Treatment of mice once daily with 78 mg/kg FMGX (plus ABT) or 104 mg/kg FMGX (plus ABT) significantly increased median survival time vs. placebo from 7 days to 13 and 11 days, respectively and enhanced overall survival by Day 21 [61]. Neither POS (30 mg/kg BID) nor liposomal AMB (L-AMB) (10 mg/kg) prolonged median survival time vs. the placebo. When tissue fungal burdens were examined, all doses of FMGX (104 to 264 mg/kg) plus ABT significantly reduced tissue fungal burden in lung and brain and were comparable to that of L-AMB treatment. In kidney, all doses of 104 mg/kg to 264 mg/kg plus ABT significantly reduced fungal burden, but only the highest dose (264 mg/kg plus ABT) demonstrated statistical significance vs. L-AMB treatment. All FMGX treatments resulted in a 2-log_10_ reduction in lung, brain, and kidney CE.

In the disseminated fusariosis model (*F. solani* 95–2478), once daily 78 mg/kg and 104 mg/kg FMGX plus ABT significantly enhanced median survival time from 7 days to 12 and 10 days, respectively [61]. Furthermore, FMGX plus ABT or L-AMB treatments equally enhanced overall survival by Day 21. Administration of a high dose of L-AMB (15 mg/kg) or FMGX (78 mg/ kg, 104 mg/kg, and 130 mg/kg) plus ABT resulted in significant reductions in kidney and brain burdens. Compared to placebo, treatment with 78, 104, or 130 mg/kg FMGX plus ABT reduced kidney counts by 2.10, 2.21, and 3.14 log_10_ CE, respectively, while L-AMB treatment resulted in a 3.96-log_10_ reduction in kidney counts. Thus, in both a pulmonary scedosporiosis model and a disseminated fusariosis model, administration of FMGX resulted in increased survival and a 2- to 3-log_10_ reduction in kidney and brain CE [61]. Reduction in tissue fungal burden was corroborated with histopathological data, with target organs showing reduced or no abscesses in FMGX-treated mice.

The efficacy of FMGX was evaluated in two pulmonary mucormycosis models using two *Rhizopus arrhizus* strains that demonstrated an 8-fold difference in MGX MEC values: 0.25 μg/mL and 4 μg/mL for *R.* *arrhizus* var. *delemar* 99–880 and *R. arrhizus* var. *arrhizus* 99–892, respectively [62]. The ISA MIC values for the two strains were 2.0 μg/mL and 1.0 μg/mL, respectively. In the *R.* *arrhizus* var. *delemar* 99–880 infection model, administration of once daily 78 mg/kg FMGX with ABT and 104 mg/kg FMGX with ABT demonstrated significantly improved survival at Day 21, similar to 110 mg/kg ISA TID, a dose which gives rise to clinically relevant exposures of ISA in mice. Tissue fungal burden was also assessed and the 78 mg/kg and 104 mg/kg FMGX dosing groups resulted in a 1.3 and 1.97 log_10_ reduction in CE/gram of lung tissue, which was similar to what was observed for ISA (1.79 log_10_ reduction in CE). Reductions in log_10_ CE/gram of brain tissue were 0.93, 1.78 and 1.65 for 78 mg/kg FMGX, 104 mg/kg FMGX, and 110 mg/kg TID ISA, respectively.

In the second *R. arrhizus* var. *arrhizus* 99–892 model (MGX MEC 4 μg/mL) both the 104 mg/kg FMGX (with ABT) or ISA (110 mg/kg TID, PO) treatments demonstrated significant efficacy (30% survival Day 21) vs. placebo control (0% survival), and the two drug treatment survival curves were not significantly different from each other. Assessment of lung and brain tissue at Day + 4 demonstrated that administration of FMGX resulted in significant reductions in lung (log_10_ 1.15) and brain (log_10_ 1.14) burden, whereas ISA reduced the fungal burden lung (log_10_ 1.69) and brain (log_10_ 1.14). ISA and FMGX CE reductions were not significantly different from each other [62]. Thus, FMGX showed significant efficacy against two strains of *Rhizopus*, with both high (4 µg/mL) and low (0.25 µg/mL) MEC values, that was as protective as ISA in two invasive pulmonary mucormycosis infection models, using highly immunosuppressed mice.

## 14. Tissue Distribution

The absorption, distribution and excretion profiles of [^14^C]FMGX-derived radioactivity were explored in rats using a single dose of 100 mg/kg PO or 30 mg/kg IV, whereas monkeys were administered a single 6 mg/kg IV dose [67]. Samples were analyzed for total radioactivity content by liquid scintillation counting, and carcasses were analyzed by quantitative whole-body autoradiography (QWBA). [14C]FMGX-derived radioactivity was rapidly and extensively absorbed and distributed to most tissues for both routes of administration in both species. In rats, tissues with the highest radioactivity Cmax values included bile, abdominal fat, reproductive fat, subcutaneous fat, and liver. Radioactivity was also detected in tissues associated with invasive fungal infections, including lung, brain and eye. In monkeys, the highest Cmax values were in bile, urine, uveal tract, bone marrow, abdominal fat, liver, and kidney cortex. Liver and kidney were the tissues with highest radioactivity. As in rats, radioactivity was also detected in lung, brain, and eye tissues. In pigmented rats, radiocarbon was densely distributed into pigmented tissue and more slowly cleared than from other tissues. Thus, FMGX-related radioactivity is extensively distributed to major tissues in both rats and monkeys and cleared primarily by biliary/fecal excretion.

## 15. Phase 1 Clinical Trials

ClinicalTrials.gov lists 4 Phase 1 clinical trials for APX001 (FMGX) including three completed studies. The first study examined the Safety, Tolerability and Pharmacokinetics of APX001 Administered Intravenously (NCT02956499). In this study six single ascending dose (SAD) and four multiple ascending dose (MAD) cohorts were evaluated and subjects were randomized in a 6:2 ratio to receive 3 h IV infusions of FMGX or placebo [64]. SAD cohorts received doses from 10–350 mg whereas MAD cohorts received doses of 50–600 mg once daily for 14 days. Analysis of the PK data showed that plasma exposures were linear and dose proportional for MGX, and low intersubject variability was observed. The half-life was ~2.5 days and accumulation of MGX was observed in the MAD cohorts, as anticipated. FMGX was well-tolerated across all doses with no clinically significant adverse events observed and there were no dose limiting toxicities. Most of the AEs were mild, transient, and required no treatment, with the most common AE being headache. The 600 mg/day AUC_0–24_ on Day 14 was 245 μg·h/mL.

The second clinical study evaluated oral administration of APX001: Safety, Pharmacokinetics, Bioavailability, Food Effect, Drug-Drug Interaction Study of APX001 Administered Orally (NCT02957929). In this study, single IV doses of 200 mg were infused over 3 h followed by single (tablet) doses of 100, 300, and 500 mg, each separated by a 14-day washout period [63]. To evaluate food effects, a single 400 mg tablet was administered under fed and fasted conditions, each separated by a 14-day washout period. Across the 4 dosing groups (100, 300, 400, and 500 mg) oral bioavailability was determined to be > 90% consistent with complete absorption and conversion to the active moiety. No food effect was observed. MAD cohorts received oral doses of 500 and 1000 mg daily for 14 days. Similar to previous observations, the MGX plasma exposure was linear and dose proportional, with low intersubject variability. FMGX was well-tolerated across all doses with no clinically significant adverse events observed and no dose limiting toxicities. Most of the AEs were mild, transient and required no treatment. After 14 days of dosing at 500 and 1000 mg AUC_0–24_ were 192 and 325 μg·/mL, respectively [63].

A third study evaluated the PK in patient populations: Safety and Pharmacokinetics of Intravenous and Oral APX001 in Patients With Acute Myeloid Leukemia (AML) and Neutropenia has completed (NCT03333005), as has a fourth study: A Drug-Drug Interaction Study of CYP3A4 Inhibition and Pan-CYP Induction on APX001 (NCT04166669). This later open-label study specifically evaluated the drug-drug interaction potential of a strong CYP3A4 inhibitor (ITC) and a pan-CYP inducer (rifampin) on FMGX in two parallel groups of healthy subjects. To date, the results of these studies have not been reported.

## 16. Phase 2 Clinical Trials

ClinicalTrials.gov lists three Phase 2 clinical trials for APX001/FMGX including one study that has completed enrollment. These three multicenter, open-label, single arm Phase 2 studies were designed to evaluate the efficacy and safety of both IV and oral FMGX for the treatment of *Candida*, *Aspergillus*, and rare molds. Enrollment has completed for the first study, An Efficacy and Safety Study of APX001 in Non-Neutropenic Patients with Candidemia (NCT03604705), in which FMGX was investigated as a first-line treatment for patients with invasive fungal infections caused by *Candida*. Patients were treated with FMGX for up to 14 days: 1000 mg intravenously twice a day for one day, then 600 mg intravenously once daily for at least two days, followed by either 600 mg intravenously once daily or 700 mg orally once daily. The primary efficacy endpoint was outcome at end of study treatment (EOST) as determined by the independent Data Review Committee (DRC). Successful outcome was defined as clearance of *Candida* from blood cultures with no additional antifungal treatment and survival at EOST. All *Candida* isolates were tested for susceptibility [68]. A key secondary efficacy endpoint was survival at Day 30. The trial enrolled 21 patients, and 20 patients were included in the modified intent-to-treat, or mITT, population. The DRC assessed 16/20 (80%) patients as treatment success at EOST. Further, 18/21 (86%) of the patients where alive at Day 30 [68]. Amplyx announced that the trial met its primary efficacy endpoint and demonstrated a treatment success rate of 80% [69]. FMGX was well-tolerated with no treatment-related serious adverse events or discontinuations.

The second study, An Open-label Study of APX001 for Treatment of Patients with Candidemia/Invasive Candidiasis Caused by *Candida auris* (NCT04148287) is currently recruiting. This study will evaluate the safety and efficacy of both intravenous and oral FMGX for the treatment of patients with candidemia, caused by *C. auris*, including patients with suspected or confirmed resistance to standard-of-care antifungal treatments. The third study, An Open-label Study of APX001 for Treatment of Patients with Invasive Mold Infections Caused by *Aspergillus* Species or Rare Molds (NCT04240886) is also currently recruiting.

## 17. Regulatory Submissions

Amplyx has received Fast Track status for IV and oral formulations of FMGX by the U.S. Food and Drug Administration for seven separate indications including treatment of invasive candidiasis, invasive aspergillosis, scedosporiosis, fusariosis, mucormycosis, cryptococcosis, and coccidioidomycosis [70]. The IV and oral formulations were previously granted Qualified Infectious Disease Product (QIDP) designations for the four qualified fungal pathogens: *Candida* spp., *Aspergillus* spp., *Coccidioides* spp., and *Cryptococcus* spp. In addition to Fast Track and QIDP designations, FMGX received orphan drug designation for the treatment of invasive candidiasis, invasive aspergillosis, cryptococcosis, coccidioidomycosis and rare mold infections caused by *Scedosporium* spp., *Fusarium* spp., and Mucorales fungi (including *Mucor* spp., and *Rhizopus* spp.).

## 18. Discussion

FMGX is a novel first-in-class antifungal in clinical development to address multiple invasive infections with high mortality. The active moiety, MGX demonstrates excellent in vitro activity against a wide range of clinically important yeasts and molds including *Candida, Aspergillus, Scedosporium, L. prolificans, Fusarium, Coccidioides*, and some other rare molds. The novel mechanism of action and broad-spectrum activity has been demonstrated to translate to efficacy in many animal models, including multi-drug resistant and other difficult-to-treat infections. This translation includes improved survival, reduction in brain, kidney, spleen, lung, and eye fungal burden, consistent with a wide tissue distribution. The reduction in tissue fungal burden has been correlated with a reduction in GM for *Aspergillus*, suggesting the potential utility of this biomarker for monitoring treatment outcomes for this fungal infection. The low frequency of resistance and lack of target-based cross-resistance to the other main classes of antifungal agents are additional attributes, especially for organisms such as *C. auris* where MDR limits treatment options. To fully understand the potential for the development of MGX resistance in the clinic, future studies are necessary to evaluate the impact of transporters, increased expression of efflux, or other as yet uncharacterized mechanisms of resistance.

Both IV and oral formulations of FMGX are currently being developed, allowing for convenient continuation of care outside of the hospital. Once daily dosing, a favorable drug-drug interaction profile with no significant CYP3A4 inhibition, and wide tissue distribution contribute to the positioning of FMGX for empiric, front-line therapy for the treatment of invasive fungal infections.

## Figures and Tables

**Table 1:** Summary of the in vitro activity of MGX against yeasts.

Organism (of Strains)	Method	MIC_90_								Reference
		MGX	FLC	VRC	POS	ITC	CAS	AFG	AMB	
*C. albicans* (21)	CLSI	0.06	64	0.5	0.5	nt	4	nt	nt	[32]
*C. albicans* (402)	EUCAST	0.016	0.25	0.008	nt	nt	nt	0.008	0.25	[34]
*C. albicans* (414)	CLSI	0.008	0.25	0.015	0.06	nt	nt	0.03	1	[30]
*C. albicans* (29)	CLSI	≤0.03	>64	nt	nt	nt	2	nt	nt	[40]
*C. auris* (16)	CLSI	0.03	>64	1	1	1	1	0.25	4	[33]
*C. auris* (122)	CLSI	0.03	512	4	0.12	0.25	nt	0.5	1	[37]
*C. auris* (200)	CLSI	0.03	256	2	0.5	1	0.25	1	2	[35]
*C. auris* (100)	CLSI	0.008	nt	nt	nt	nt	nt	nt	nt	[41]
*C. dubliniensis* (48)	EUCAST	0.008	0.5	0.016	nt	nt	nt	0.016	0.06	[34]
*C. dubliniensis* (49)	CLSI	0.008	0.25	0.015	0.06	nt	nt	0.12	0.06	[30]
*C. glabrata* (321)	CLSI	0.12	32	1	1	nt	nt	0.12	1	[30]
*C. glabrata* (20)	CLSI	0.06	32	1	1	nt	2	nt	nt	[32]
*C. glabrata* (188)	EUCAST	0.125	32	0.5	nt	nt	nt	0.03	0.5	[34]
*C. kefyr* (12)	EUCAST	0.5	0.5	0.016	nt	nt	nt	0.03	0.5	[34]
*C. kefyr* (13)	CLSI	0.5	0.25	≤0.008	0.12	nt	nt	0.12	1	[30]
*C. krusei* (43)	CLSI	>2	32	0.5	0.5	nt	nt	0.12	1	[30]
*C. krusei* (54)	EUCAST	>0.5	64	0.5	nt	nt	nt	0.06	1	[34]
*C. lusitaniae* (39)	CLSI	0.12	2	0.015	0.12	nt	nt	0.5	1	[30]
*C. lusitaniae* (12)	EUCAST	0.016	0.5	0.016	nt	nt	nt	0.06	0.25	[34]
*C. parapsilosis* (270)	CLSI	0.015	2	0.06	0.12	nt	nt	1	1	[30]
*C. parapsilosis* (25)	CLSI	0.06	16	0.25	0.12	nt	4	nt	nt	[32]
*C. parapsilosis* (39)	EUCAST	0.03	2	0.03	nt	nt	nt	2	0.5	[34]
*C. tropicalis* (24)	CLSI	0.06	64	2	0.5	nt	4	nt	nt	[32]
*C. tropicalis* (151)	CLSI	0.03	0.5	0.06	0.5	nt	nt	0.06	1	[30]
*C. tropicalis* (41)	EUCAST	0.016	1	0.03	nt	nt	nt	0.03	0.5	[34]
*C. neoformans* (30)	CLSI	0.5	4	0.06	0.25	nt	nt	>4	1	[30]

**Table 2:** CLSI MGX MIC values vs. collections of *C. auris* strains.

Strain Source	Strains	MIC_50_ (µg/mL)	MIC_90_ (µg/mL)	MIC Range (µg/mL)	Reference
Worldwide	100	0.004	0.008	<0.0005–0.015	[41]
India, Japan, Korea, Germany	16	0.004	0.03	0.002–0.015	[33]
Worldwide	13	0.03	0.03	≤0.002–0.03	[43]
USA	1	--	--	0.06	[30]
USA	5	0.03	--	0.03–0.06	[28]
Panama	6	0.004	--	≤0.002–0.015	[28]
India	122	0.008	0.03	0.001–0.25	[37]
NY/NJ, USA	200	0.016	0.03	0.008 -0.03	[35]

**Table 3:** Summary of the in vitro activity of MGX against molds.

Organism (of Strains)	Method	MIC_90_/MEC_90_							Reference
		MGX	VRC	POS	ITC	CAS	AFG	AMB	
*Alternaria alternata* (10)	CLSI	4	nt	16	nt	nt	nt	4	[31]
*A. alliaceus* (10)	CLSI	0.03	nt	0.12	nt	nt	nt	32	[31]
*A. calidoustus* (10)	CLSI	0.03	nt	16	nt	nt	nt	1	[31]
*A. flavus* (20)	CLSI	0.03	1	1	1	0.12	≤0.008	nt	[44]
*A. flavus* (18)	CLSI	0.015	1	0.5	1	nt	0.03	2	[30]
*A. fumigatiaffinis* (10)	CLSI	0.03	nt	0.5	nt	nt	nt	4	[31]
*A. fumigatus* (182)	CLSI	0.03	0.5	0.5	1	nt	0.03	2	[30]
*A. fumigatus* (22)	CLSI	0.06	2	1	>8	0.12	0.015	nt	[44]
*A. fumigatus* (121)	EUCAST	0.06	0.5	0.12	0.25	nt	nt	0.5	[29]
*A. lentulus* (10)	CLSI	0.03	nt	1	nt	nt	nt	1	[31]
*A. niger* (18)	EUCAST	0.03	2	0.25	1	nt	nt	0.25	[29]
*A. niger* (13)	CLSI	0.015	2	1	4	0.12	≤0.008	nt	[44]
*A. niger* (23)	CLSI	0.015	2	1	4	nt	0.015	1	[30]
*A. terreus* (23)	CLSI	0.06	1	0.5	1	0.12	0.015	nt	[44]
*A. terreus* (10)	CLSI	0.03	0.5	0.25	0.5	nt	0.015	2	[30]
*A. thermomutatus* (10)	CLSI	0.25	nt	1	nt	nt	nt	2	[31]
*A. udagawae* (10)	CLSI	0.06	nt	0.5	nt	nt	nt	1	[31]
*Cunninghamella bertholletiae* (10)	CLSI	16	nt	2	nt	nt	nt	4	[31]
*F. solani* (15)	CLSI	0.06	>8	>8	>8	>8	>8	2	[36]
*F. oxysporum* (15)	CLSI	0.25	4	2	>8	>8	>8	4	[36]
*F. oxysporum* (10)	CLSI	16	nt	16	nt	nt	nt	1	[31]
*F. verticilloides* (10)	CLSI	16	nt	16	nt	nt	nt	32	[31]
*Gibberella fujikuroi* (30)	CLSI	0.12	8	>8	>8	>8	>8	4	[36]
*Lichtheimia corymbifera* (10)	CLSI	16	nt	2	nt	nt	nt	0.12	[31]
*Lichtheimia ramosa* (10)	CLSI	16	nt	0.5	nt	nt	nt	0.06	[31]
*Lomentospora prolificans* (10)	CLSI	0.06	nt	16	nt	nt	nt	8	[31]
*M. circinelloides* (10)	CLSI	8	nt	1	nt	nt	nt	0.03	[31]
*Rhizomucor pusillus* (10)	CLSI	16	nt	2	nt	nt	nt	0.06	[31]
*R. arrhizus* (10)	CLSI	16	nt	0.5	nt	nt	nt	0.12	[31]
*R. microsporus* (10)	CLSI	16	nt	2	nt	nt	nt	0.12	[31]
*S. apiospermum* (28)	CLSI	0.12	1	2	4	>8	4	>4	[36]
*S. apiospermum* (10)	CLSI	16	nt	8	nt	nt	nt	32	[31]
*S. aurantiacum* (10)	CLSI	0.03	nt	16	nt	nt	nt	16	[31]
*S. boydii* (10)	CLSI	0.12	nt	2	nt	nt	nt	2	[31]
*S. prolificans* (28)	CLSI	0.12	>8	>8	>8	>8	4	>4	[36]

**Table 4:** Summary of animal efficacy models for manogepix and fosmanogepix.

Pathogen	Infection Type	Efficacy Endpoint (Special Study)	Reference
**Manogepix**			
*C. albicans*	oropharyngeal	Oral CFU	[53]
*C. albicans*	disseminated	Survival	[53]
*C. albicans*	disseminated	Survival, kidney CFU	[40]
*C. tropicalis*	disseminated	Survival	[53]
*A. fumigatus*	pulmonary	Survival	[53]
*A. flavus*	pulmonary	Survival	[53]
*F. solani*	disseminated	Survival	[53]
**Fosmanogepix**			
*C. albicans*	oropharyngeal	Oral CFU	[16]
*C. albicans*	disseminated	Survival	[16]
*C. albicans*	disseminated	Kidney CFU	[51]
*C. albicans*	disseminated	Kidney CFU (PK/PD)	[54]
*C. albicans* ^1^	disseminated	Brain, eye CFU	[55]
*C. auris*	disseminated	Survival; kidney, lung, brain CFU	[33]
*C. auris*	disseminated	Survival; kidney, lung, brain CFU	[43]
*C. auris*	disseminated	Kidney CFU (PK/PD)	[54]
*C. glabrata*	disseminated	Kidney CFU (PK/PD)	[54]
*C. glabrata*	disseminated	Kidney CFU	[51]
*C. immitis*	pulmonary	Survival; lung, spleen CFU	[47]
*C. neoformans*	disseminated	Brain, lung CFU	[56]
*A. flavus*	pulmonary	Survival	[16]
*A. flavus*	pulmonary	Survival (combination MFG, AFG)	[57]
*A. fumigatus*	pulmonary	Survival	[16]
*A. fumigatus*	pulmonary	Lung CFU (PK/PD)	[58]
*A. fumigatus*	pulmonary	Lung CFU, survival, histology	[59]
*A. fumigatus*	pulmonary	Lung CFU; serum, BAL GM (GM biomarker)	[60]
*F. solani*	disseminated	Survival	[16]
*F. solani*	disseminated	Survival; kidney, brain CFU; histology	[61]
*R. arrhizus var. arrhizus*	pulmonary	Survival; lung, brain CFU	[62]
*R. arrhizus var. delemar*	pulmonary	Survival; lung, brain CFU	[62]
*S. apiospermum*	pulmonary	Survival; kidney, lung, brain CFU; histology	[61]
*S. prolificans*	pulmonary	Survival	[16]

^1^ Non-neutropenic rabbit model of *Candida* endophthalmitis and hematogenous meningoencephalitis.
